# Predictors of Pediatric COVID-19 vaccination: a case-control study in Tabriz, Iran

**DOI:** 10.1186/s12887-023-04202-y

**Published:** 2023-07-31

**Authors:** Parvin Sarbakhsh, Nasrin Jafari, Saman Salemi, Reza Akbarnejad

**Affiliations:** 1 Department of Statistics and Epidemiology, Faculty of Health, Tabriz University of Medical Sciences, Tabriz, Iran; 2 Student Research Committee, Tabriz University of Medical Sciences, Tabriz, Iran; 3 Department of Medicine, Islamic Azad University Tehran Medical Sciences, Tehran, Iran; 4 Department of Knowledge and Information Science, Education and Psychology Faculty, Azarbaijan Shahid Madani University, Tabriz, Iran

**Keywords:** Child, COVID-19 vaccines, Vaccination hesitancy, Socioeconomic factors

## Abstract

**Introduction:**

COVID-19 vaccination of children can help reduce the severity of the infection and the death rate caused by it and also helps achieve herd immunity. The level of acceptance and high vaccination coverage is the main elements in the success of immunization programs. Children’s vaccination is dependent on their parent’s decision. This study aims to identify predictors of the children’s COVID-19 vaccination accomplishment by their parents.

**Method:**

In this case-control study, 577 vaccinated children as cases and 366 un-vaccinated children as controls were randomly selected from the general population of Tabriz, Iran 2022, and their data were collected by telephone calls and interviews with the children’s parents. Cases and controls were compared in terms of clinical and demographic factors of the child as well as the socioeconomic status (SES) of their parents by using a multivariable mixed-effect logistic regression model.

**Results:**

According to the results of the multivariable logistic regression, the age of the child (OR = 1.26 95% CI (1.14, 1.40), p-value < 0.001), previous COVID-19 infection of the child (OR = 1.92, 95% CI (1.21, 3.04), p-value < 0.001), having no underlying disease in the child (OR = 1.76, 95% CI (1.02, 3.02), p-value = 0.04), the dwelling place of the household (the high-level dwelling in compared to a low level (OR = 3.34, 95% CI (1.6, 6.64), p-value = 0.001), the middle level of dwelling compared with low level (OR = 4.87, 95% CI (2.46, 9.51), p-value < 0.001)), and Father’s job (Employee and technician Fathers compared to worker fathers (OR = 2.99, 95% CI (1.55, 5.77), p-value = 0.001)) were significant independent predictors of children COVID-19 vaccination.

**Conclusion:**

Several demographic and socioeconomic factors were associated with children’s vaccination. Older children, children without any underlying disease, children with a history of COVID-19 infection, and children of parents with higher levels of SES were more likely to receive the COVID-19 vaccine. This finding can be considered in children’s vaccination policymaking.

## Introduction

COVID-19 vaccination, as one of the most successful and cost-effective public health interventions, can help reduce the severity of the infection and the death rate caused by it and also helps achieve herd immunity [1]. However, despite the safety and effectiveness of immunization measures, vaccination hesitancy (delay in accepting vaccination despite the availability of an effective and safe vaccine) has become an emerging global issue [2, 3].

The successful implementation of the vaccination program is not limited to the vaccine’s efficiency and effectiveness. In addition to the existence of a coherent and strong health system, there is an urgent need for public trust and acceptance of the target community towards the vaccine. Previous experiences in the world and Iran confirm that the level of acceptance and high coverage is the main elements in the success of immunization programs. However, based on the evidence, there are concerns and doubts about vaccination among people in different countries.

In the case of coronavirus disease 2019 (COVID-19), hesitance to accept the COVID-19 vaccine can seriously undermine global efforts to control the current pandemic. It will affect and impose more life and economic burden on human societies, increasing the problems in countries with limited resources.

Hesitation and rejection of COVID-19 vaccination in adults are influenced by many factors such as demographic, political, cultural, socioeconomic factors, fear, and fatalism confusion caused by the abundance of information, mistrust, as well as concern about the side effects of the vaccines [4–10].

Regarding children’s COVID-19 vaccination, Iran started to vaccinate children aged 5 to 12 years on February 8, 2022, with Sinopharm and Soberana (PastoCoVac) vaccines which were injected on two occasions with an interval of at least 28 days. Vaccination services in Iran are free of charge under the supervision of medical sciences universities, healthcare centers, and mass vaccination centers. According to the formal reports of Iran’s Ministry of Health, the number of COVID-19 vaccination doses administered per 100 people in Iran rose to 175 as of April 29, 2023. The coverage rate of two dose-vaccination for Iranian people aged > 12 years is more than 75%, but this rate is a maximum of 10% for children aged 5–12.

According to the studies, the acceptance rate of COVID-19 vaccination for children worldwide ranges from 20 to 80% [11–14]; children are not decision-makers regarding vaccination, and this decision depends on their parents. Evaluating the factors that may predict parents’ inclination towards vaccinating their children is crucial. Parental acceptance of COVID-19 vaccination for children was associated with several factors, such as place, time, culture, religion, and race. Previous studies in Iran and other countries have reported some associated factors such as vaccine literacy [15, 16], attitudes regarding vaccines [16], trust in scientists [13], Parents’ hesitancy to receive the COVID-19 vaccine for themselves [11], wrong beliefs [17] concerns about vaccine efficacy and safety [18], demographic characteristics, and social-economic status (SES) of parents [16, 19, 20].

As far as current research indicates, no studies have been reported on the factors influencing parental acceptance of childhood vaccinations in Tabriz, Iran. Considering the ethnic and cultural distinctions between Tabriz and other cities in Iran, conducting this research in Tabriz appears worthwhile. Furthermore, while clinical and demographical characteristics of the child can also affect the decision of parents to do vaccination, most studies have investigated just effects of parents’ features such as knowledge and attitude toward vaccines, demographic, and SES characteristics on children’s vaccination. Also, most of the studies had cross-sectional descriptive designs that assessed acceptance (willingness to take a vaccine if proven safe and effective), and no study evaluated their practice regarding vaccination.

So, the current study aims to assess the association of demographic and clinical features of the child as well as SES of the parents with the accomplishment of the COVID-19 vaccination in children aged 5 to 12 years of Tabriz in 2022 with a case-control design and compare vaccinated and unvaccinated children according to their vaccination-related variables.

## Materials and methods

### Study design

This case-control study is a follow-up to a primary study conducted in Tabriz, Iran, from March 2022 to September 2022 to assess the COVID-19 vaccination complications in children. The report of the original study has been submitted and is under review [21].

The cluster sampling method was used for data collection in the primary study. Thus, five healthcare facilities and one mass vaccination center were chosen randomly from 20 healthcare facilities and three mass vaccination centers for COVID-19.

A ratio of 2:1 was considered in the sample size calculation of the vaccinated and unvaccinated groups, respectively. The required sample size was estimated using the sample size formula for comparing two independent proportions. Considering the 3% prevalence of fever as the main symptom in the control group, and expecting 8% in the vaccinated group (a 5% difference between the two groups), 5% of type I error, 80% of statistical power, and a value of 1.2 for the design effect of cluster sampling, sample sizes of 617 in the vaccinated group and 309 in the control group were calculated. In practice, we could finally investigate 913 subjects aged 5 to 12 years, including 577 (63%) vaccinated and 336 (37%) unvaccinated children due to the decrease in the vaccination rate.

### Participants

The parents of qualified children comprised our sample. We collected the necessary data about the parents and their children through phone calls. The health integrated registration system (SIB) used to register all primary health care services and vaccinations in Iran was used to recruit study participants in selected centers.

Our inclusion criteria for the vaccinated group were: parents of children aged 5 to 12 who received first, second, or later doses of the COVID-19 vaccine one week ago, and for the unvaccinated group were: parents of children aged 5 to 12 who had not received any dose of the COVID-19 vaccine yet. Our exclusion criteria for both groups were the inability to call parents and the reluctance of parents to participate in the study.

The list of children from the selected centers was extracted through the SIB registry system. From children who visited selected health centers or collective centers and received the COVID-19 vaccine, 577 children were randomly selected, and their parents were asked to participate in the study (as the vaccinated group). The list of children aged 5 to 12 who received no COVID-19 vaccine was also extracted from the SIB system, and 366 were randomly selected as the control group. The sampling ratio was almost 2:1, and during the study period, gradually, for every 2 cases (children vaccinated one week ago), approximately one control was selected simultaneously. They were matched for the time of the study, which has importance in terms of peaks and variations of COVID-19 that could affect parents’ decisions for children’s vaccination.

Cases and controls were representative of the general population of Tabriz. After calling their parents by telephone and receiving verbal informed consent, they were included in the study and interviewed by phone.

### Data collection

Data were collected by using a researcher-made checklist. In both groups, information about children’s age, height, and weight, underlying disease history, history of the COVID-19 infection, parents’ education, occupation, and place of residence was obtained. We compared the demographic, clinical, and socioeconomic variables between vaccinated and unvaccinated children. The current study was approved by the ethics committee of Tabriz University of Medical Sciences (ethics code: IR.TBZMED.REC.1402.121). After explaining the purpose of the present study and obtaining verbal informed consent over the phone from the children’s parents, they entered the study. Parents who did not agree to continue the telephone interview were excluded from the study.

### Statistical analysis

Descriptive statistics of the study variables were reported by mean (SD) for normally distributed variables and number (%) for categorical variables. An Independent t-test compared the two groups according to the interested continuous variables. The Pearson chi2 or Fisher exact test was employed to compare categorical variables. A multivariable mixed-effect logistic regression was conducted to examine the adjusted effect of predictors while accounting for the cluster sampling design. Two-tailed tests with a significance level of 0.05 were conducted for data analysis. Data analysis was done using STATA 18 software.

## Results

We investigated data form 913 subjects aged 5 to 12, including 577 (63.2%) vaccinated and 336 (36.8%) unvaccinated children. Table 1 shows the background characteristics of the enrolled participants.

**Table 1:** Background characteristics of the participants

Variable	Vaccinated Children (n = 577)	Unvaccinated children (n = 336)	P-value
**Gender, n(%)**			0.680*
Male	275(47.7)	155 (46.1)	
Female	302 (52.3)	181 (53.9)	
**Age, Mean (SD)**	8.9 (1.9)	7.7 (2.0)	< 0.001^¥^
**BMI, Mean (SD)**	17.87 (3.88)	16.29 (3.14)	< 0.001^¥^
**History of COVID-19 (Yes), n(%)**	196(34.0)	58 (17.3)	< 0.001*
**Diabetes type 1 (Yes), n(%)**	0 (0.0)	2 (0.6)	0.13^£^
**Asthma (Yes), n(%)**	11 (1.9)	9 (2.7)	0.44*
**Hemoglobinopathies (Yes), n(%)**	10 (1.7)	14 (4.2)	0.03*
**Convulsion (Yes), n(%)**	9 (1.6)	4 (1.2)	0.77^£^
**Allergy (Yes), n(%)**	14 (2.4)	13 (3.9)	0.21*
**Other diseases (Yes), n(%)**	10 (1.7)	12 (3.6)	0.08*
**Level of Dwelling place, n(%)**			< 0.001*
High Level	109 (20.0)	28 (8.3)	
Middle Level	137 (25.1)	17 (5.1)	
Low Level	300 (54.9)	291 (86.6)	
**Father Education, n(%)**			< 0.001*
High school and lower	186 (33.1)	164 (50.5)	
Undergraduate	284 (50.5)	146 (44.9)	
Graduate (Masters, PhD)	92 (16.4)	15 (4.6)	
**Mother Education, n(%)**			< 0.001*
High school and lower	166 (29.5)	170 (51.8)	
Undergraduate	350 (62.2)	144 (43.9)	
Graduate (Masters, PhD)	47 (8.3)	14 (4.3)	
**Father job, n(%)**			< 0.001*
Managers and specialists	42 (7.8)	11 (3.4)	
Employees and technicians	161 (29.8)	33 (10.2)	
Craftsmen and sellers	248 (45.8)	169 (502.2)	
Workers	90 (16.6)	110 (34.3)	
**Mother job, n(%)**			< 0.001*
Managers and specialists	23 (4.2)	4 (1.2)	
Employees and technicians	51 (9.4)	14 (4.3)	
Craftswomen and sellers	12 (2.1)	18 (5.5)	
Housewife	459 (84.2)	291 (89.0)	

Descriptive statistics for all variables were calculated from valid cases

*P-value was calculated by Chi^2^ test

^¥^ P-value was calculated by independent T-test

^£^ P-value was calculated by the Fisher exact test

In univariate analysis, significant differences (p-value < 0.05) were observed between the case and control groups for all underlying demographic, clinical, and socioeconomic variables except for the sex of the child (Table 1). Compared to controls, cases were older (8.9 ± 1.9 years vs. 7.7 ± 2.0 years), had higher BMI (17.87 vs. 16.29 kg/m2), had less underlying disease rate (9.4% vs. 15.9%), and had a higher rate of previous COVID-19 infection (34.05 vs. 17.3%). Of cases, 20.0% were in the high level of dwelling status (vs. 8.3% of controls), 77% of cases’ fathers (vs. 50.5 controls), and 69.5% of cases’ mothers (vs. 48.55) had academic education. Regarding parents’ jobs, compared with the controls, cases had a higher percentage of manager, specialist, employee, or technician fathers (48.3% vs. 15.7%) and mothers (13.6% vs. 5.5%).

According to the results of the multivariable mixed effect logistic regression, age (OR = 1.26, 95% CI(1.14, 1.40), p-value < 0.001), child’s previous COVID-19 infection (OR = 1.92, 95% CI(1.21, 3.04), p-value < 0.001), having no underlying disease (OR = 1.76, 95% CI(1.02, 3.02), p-value = 0.04), dwelling place (high-level dwelling place in compared to the low level (OR = 3.34, 95% CI(1.68, 6.64), p-value = 0.001), middle dwelling place compared with low level (OR = 4.87, 95% CI(2.46, 9.51), p-value < 0.001)), and father’s job (Employee and technician Fathers compared to worker fathers (OR = 2.99, 95% CI(1.55, 5.77), p-value = 0.001)) were significant independent predictors of children vaccine acceptance by their parents (Table 2).

**Table 2:** Multivariable mixed effect logistic regression to assess the association between characteristics of the participants and vaccine acceptance

Variable	Adjusted OR	95% CI for OR		P-value
		lower	upper	
**Gender**				
Male	1.09	0.75	1.58	0.628
Female	Referent			-*
**Age**	1.26	1.14	1.40	**< 0.001**
BMI	1.036	0.98	1.09	0.211
**History of COVID-19 infection**				
yes	1.92	1.21	3.04	0.005
no	Referent			-*
**Having any Underlying Diseases**				
no	1.76	1.02	3.02	0.04
yes	Referent			-*
**Level of Dwelling place**				
High Level	3.34	1.68	6.64	0.001
Middle Level	4.87	2.49	9.50	< 0.001
Low Level	Referent			-*
**Father Education**				
High school and lower	Referent			-*
Undergraduate	0.90	0.56	1.44	0.669
Graduate (Masters, PhD)	1.27	0.48	3.32	0.621
**Mother Education**				
High school and lower	Referent			-*
Undergraduate	1.21	0.77	1.92	0.396
Graduate (Masters, PhD)	0.90	0.29	2.73	0.853
**Father job**				
Managers and specialists	1.67	0.57	4.89	0.348
Employees and technicians	2.99	1.55	5.77	**0.001**
Craftsmen and sellers	1.53	0.98	2.40	0.059
Workers	Referent			-*
**Mother job**				
Managers and specialists	1.04	0.25	4.28	0.955
Employees and technicians	0.70	0.27	1.80	0.465
Craftswomen and sellers	0.68	0.26	1.76	0.433
Housewife	Referent			-*

*Reference group

## Discussion

Regarding children’s vaccination, parents are decision-makers, and identifying predictors of this decision has high importance for successful vaccination programs. This study aimed to assess the association of clinical and demographic factors of the child as well as the household’s socioeconomic status with their parents’ acceptance and accomplishment of the vaccination. To do this, we assessed data from 577 vaccinated and 366 un-vaccinated children in this case-control study. Vaccinated and un-vaccinated children were compared in terms of some demographic, clinical, and SES characteristics of the children and their parents.

According to the results of a multivariable logistic regression, several demographic and socioeconomic factors were associated with parents’ inclination to their children’s vaccination. Children who were older, healthier, had a family history of COVID-19, or came from families with a higher household income were more likely to receive the vaccine.

Previous studies indicated that adult vaccination hesitation is influenced by various factors, such as demographic, political, cultural, and socioeconomic factors, attitude, knowledge, safety, trust, fear, and fatalism confusion caused by the abundance of information and the connection between vaccination and some diseases [5, 7–10]. Also, several studies have evaluated the relationship between the SES of people with the acceptance and hesitancy of the COVID-19 vaccination in different populations [6, 8, 9, 22].

The reluctance of parents to vaccinate their children against COVID-19 is a multifaceted issue that can be influenced by various factors such as cultural background, ethnicity, income level, and health policies. These predictors may have varying effects on vaccine acceptance depending on the context. There are various studies regarding parental COVID-19 vaccination hesitancy. Most have studied the effect of knowledge, attitude, and trust toward vaccines on vaccination hesitancy. Mollaie et al. evaluated the role of wrong beliefs on vaccination hesitancy. They concluded that most parents believed that COVID-19 vaccines have side effects for their children and unfavorable effects on children’s growth and infertility [17].

Alimoradi et al. conducted a systematic review and meta-analysis study to assess parental acceptance of children COVID-19 vaccine. They showed that parents’ COVID-19 vaccine knowledge, trust in the COVID-19 vaccine, and facilitators in vaccination were significant predictors of higher willingness toward children’s vaccination, while mental health problems were significant factors for lower willingness [23].

Regarding parental vaccination acceptance, our adjusted model results indicated that age, underlying disease, and history of COVID-19 infection in children, besides SES (including the level of dwelling place and the father’s job), were significant predictors of parents’ decision for children vaccination. Regarding age, underlying disease, and a child’s history of COVID-19 infection, the results are reasonable and show more parents’ caution for younger children and children with underlying disease. Also, parents of children with a history of COVID-19 infection were more likely to be vaccination acceptance, maybe for fear of re-infection of their child.

Regarding age, consistent with our results, Zhang et al. indicated that being a child under 18 years old was a significant predictor of parental vaccine hesitancy [24]. Regarding clinical factors, including underlying disease and history of COVID-19 infection, we could not find any similar studies to compare with the current study.

Furthermore, according to our multivariable analysis results, parents with higher levels of SES factors were more likely to do vaccination for their children, which is consistent with various previous studies. They have demonstrated that household financial well-being can predict vaccine hesitancy for their children [16, 19, 20]. Nevertheless, inconsistent with our results, Lu et al.‘s [25] findings showed that Chinese parents with less than average income had lower hesitancy toward children’s vaccination against COVID-19. According to Swanery et al. [26], Australian parents with a higher SES status exhibited greater vaccine hesitancy due to reasons such as a reluctance to exert control over their children’s health decisions. The individuals’ considerations revolve around ensuring the safety of their families from diseases, evaluating the potential risks associated with vaccines, and having faith in the protective impact of their lifestyle against illnesses that can be prevented by vaccines.

To investigate how SES affects vaccine acceptance, previous studies showed that the attitude and knowledge of parents about vaccination are important predictors of the willingness of parents to get their children vaccinated against COVID-19 [15, 16, 27–29]. These variables are mediators, and SES characteristics such as job, education, and household economic level can affect acceptance of vaccination (positively or negatively) through the attitude and knowledge of parents regarding vaccination for themselves and their children.

In this regard, Sharifinia et al. conducted a multi-country study to investigate the mediating role of parent attitudes toward COVID-19 vaccines in the association path between SES and parental vaccine hesitancy. Although the direction of this association was different depending on the country, their results indicated a significant mediating role of parent attitudes toward vaccines in the link between perceived financial well-being and parental vaccine hesitancy [30].

To the researchers’ knowledge, it is the first study in Tabriz that evaluates the predictors of children’s COVID-19 vaccination which would be valuable because of the various cultural and ethnic differences between Tabriz and other cities in Iran. Furthermore, while beside the factors related to parents, such as knowledge, attitude, and fear of side effects, other clinical and demographical characteristics related to the child can also be contributed to his parent’s decision on vaccination, most studies have examined just parents’ characteristics; we assessed the effects of both child and parents’ characteristics on children’s vaccine uptake. Also, most studies had a cross-sectional descriptive design that just assessed acceptance of vaccination, not performing of vaccination; these gaps were covered in our study by the case-control design of the study and compared vaccinated and unvaccinated children according to their vaccination-related variables.

### Limitations

in the primary study, data collection was done by phone interview; this issue can affect the precision of the data. Furthermore, the self-reported nature of gathered clinical and SES variables can affect data accuracy, which would be one of our study’s limitations. Also, we could not gather the estimated sample size of vaccinated children due to the decrease in the vaccination rate. Another limitation of our study is that due to the phone interview nature of the study, we assessed the economic status of the household just by asking about the level of the dwelling place and did not use any other SES questionnaire.

## Conclusion

Overall, COVID-19 vaccine hesitancy, especially in children, is a prevalent global issue with various determinants. In this case-control study, we compared vaccinated and unvaccinated children according to some demographic, clinical, and SES variables to determine related factors to children’s COVID-19 vaccine uptake. The results demonstrated that older children, children without underlying disease, children with a history of COVID-19 infection, and children with better SES were more likely to uptake the COVID-19 vaccine. Healthcare providers and policymakers must be aware of these factors when formulating children’s COVID-19 vaccination policies.

## Data Availability

The data that support the findings of this study are available from the corresponding author upon reasonable request.

## References

[1] Scobie HM, Johnson AG, Suthar AB, Severson R, Alden NB, Balter S (2021). Monitoring incidence of COVID-19 cases, hospitalizations, and deaths, by vaccination status—13 US jurisdictions, April 4–July 17, 2021. Morb Mortal Wkly Rep.

[2] Pormohammad A, Zarei M, Ghorbani S, Mohammadi M, Razizadeh MH, Turner DL (2021). Efficacy and safety of COVID-19 vaccines: a systematic review and meta-analysis of randomized clinical trials. Vaccines.

[3] Attwell K, Lake J, Sneddon J, Gerrans P, Blyth C, Lee J (2021). Converting the maybes: crucial for a successful COVID-19 vaccination strategy. PLoS ONE.

[4] Shih SF, Wagner AL, Masters NB, Prosser LA, Lu Y, Zikmund-Fisher BJ (2021). Vaccine hesitancy and rejection of a vaccine for the Novel Coronavirus in the United States. Front Immunol.

[5] Rellosa N (2022). COVID-19 vaccine hesitancy and refusal:: the same but different?. Del J public health.

[6] Miner CA, Timothy CG, Percy K, Mashige, Osuagwu UL, Envuladu EA (2023). Acceptance of COVID-19 vaccine among sub-saharan Africans (SSA): a comparative study of residents and diasporan dwellers. BMC Public Health.

[7] Cerda AA, García LY. Hesitation and Refusal Factors in Individuals’ Decision-Making Processes Regarding a Coronavirus Disease 2019 Vaccination. Front Public Health. 2021;9. 10.3389/fpubh.2021.626852 PMC8096991 33968880

[8] Salimi Y, Paykani T, Ahmadi S, Shirazikhah M, Almasi A, Biglarian A (2021). Covid-19 Vaccine Acceptance and its related factors in the General Population of Tehran and Kermanshah. Iran J Epidemiol.

[9] Lindholt MF, Jørgensen F, Bor A, Petersen MB (2021). Public acceptance of COVID-19 vaccines: cross-national evidence on levels and individual-level predictors using observational data. BMJ Open.

[10] Roy DN, Biswas M, Islam E, Azam MS (2022). Potential factors influencing COVID-19 vaccine acceptance and hesitancy: a systematic review. PLoS ONE.

[11] Deng J-S, Chen J-Y, Lin X-Q, Huang C-L, Tung T-H, Zhu J-S (2023). Parental hesitancy against COVID-19 vaccination for children and associated factors in Taiwan. BMC Public Health.

[12] Bell S, Clarke R, Mounier-Jack S, Walker JL, Paterson P (2020). Parents’ and guardians’ views on the acceptability of a future COVID-19 vaccine: a multi-methods study in England. Vaccine.

[13] Baumann BM, Rodriguez RM, DeLaroche AM, Rayburn D, Eucker SA, Nadeau NL (2022). Factors Associated with parental Acceptance of COVID-19 vaccination: a Multicenter Pediatric Emergency Department cross-sectional analysis. Ann Emerg Med.

[14] Lee H, Choe YJ, Kim S, Cho H-K, Choi EH, Lee J (2022). Attitude and Acceptance of COVID-19 vaccine in parents and adolescents: a Nationwide Survey. J Adolesc Health.

[15] Lee M, Seo S, Choi S, Park JH, Kim S, Choe YJ (2022). Parental Acceptance of COVID-19 vaccination for children and its Association with Information Sufficiency and credibility in South Korea. JAMA Netw Open.

[16] Maneesriwongul W, Butsing N, Deesamer S (2023). Parental hesitancy on COVID-19 vaccination for children under five years in Thailand: role of attitudes and vaccine literacy. Patient Prefer Adherence.

[17] Mollaie M, Mirahmadizadeh A, Sanaei Dashti A, Jalalpour AH, Jafari K (2023). COVID-19 vaccination hesitancy among 5–11-year-old iranian children’s parents: what are underlying beliefs?. Arch Pediatr Infect Dis.

[18] Kheir-Mataria AE, Saleh W, El-Fawal BM, Chun H. S. COVID-19 vaccine hesitancy among parents in low- and Middle-Income Countries: a meta-analysis. Front Public Health. 2023;11. 10.3389/fpubh.2023.1078009 PMC10010145 36923043

[19] Mohan R, Pandey V, Kumar A, Gangadevi P, Goel AD, Joseph J (2022). Acceptance and attitude of parents regarding COVID-19 vaccine for children: a cross-sectional study. Cureus.

[20] Maharlouei N, Hosseinpour P, Erfani A, Shahriarirad R, Raeisi Shahrakie H, Rezaianzadeh A (2022). Factors associated with reluctancy to acquire COVID-19 vaccination: a cross-sectional study in Shiraz, Iran, 2022. PLoS ONE.

[21] Jafari N, Akbari H, Khayatzadeh S, Nobakht H, Sarbakhsh P. Covid-19 vaccination complications in children: a prospective investigation with a Control Group in Tabriz Metropolitan City. Vaccine Res. 2023; submitted, under review.

[22] Mirahmadizadeh A, Mehdipour Namdar Z, Miyar A, Maleki Z, Hashemi Zadehfard Hagheghe L, Sharifi MH (2022). COVID-19 Vaccine Acceptance and its risk factors in Iranian Health Workers 2021. Iran J Med Sci.

[23] Alimoradi Z, Lin C-Y, Pakpour AH (2023). Worldwide Estimation of parental Acceptance of COVID-19 vaccine for their children: a systematic review and Meta-analysis. Vaccines.

[24] Zhang MX, Lin XQ, Chen Y, Tung TH, Zhu JS (2021). Determinants of parental hesitancy to vaccinate their children against COVID-19 in China. Expert Rev Vaccines.

[25] Lu J, Wen X, Guo Q, Ji M, Zhang F, Wagner AL et al. Sensitivity to COVID-19 Vaccine Effectiveness and Safety in Shanghai, China. Vaccines (Basel). 2021;9(5). 10.3390/vaccines9050472 PMC8151750 34067141

[26] Swaney SE, Burns S (2019). Exploring reasons for vaccine-hesitancy among higher-SES parents in Perth, Western Australia. Health Promot J Austr.

[27] Juin JCY, Ern SLS, Min C, Jing NK, Qi MNM, Hoe RCC (2022). Knowledge, attitudes, and Practices of COVID-19 vaccination among adults in Singapore: a cross-sectional study. Am J Trop Med Hyg.

[28] Sherman SM, Smith LE, Sim J, Amlôt R, Cutts M, Dasch H (2021). COVID-19 vaccination intention in the UK: results from the COVID-19 vaccination acceptability study (CoVAccS), a nationally representative cross-sectional survey. Hum vaccines immunotherapeutics.

[29] Islam MS, Siddique AB, Akter R, Tasnim R, Sujan MSH, Ward PR (2021). Knowledge, attitudes and perceptions towards COVID-19 vaccinations: a cross-sectional community survey in Bangladesh. BMC Public Health.

[30] Sharif Nia H, Allen K-A, Arslan G, Kaur H, She L, Khoshnavay Fomani F et al. The predictive role of parental attitudes toward COVID-19 vaccines and child vulnerability: a multi-country study on the relationship between parental vaccine hesitancy and financial well-being. Front Public Health. 2023;11. 10.3389/fpubh.2023.1085197 PMC9980903 36875362

